# Polyphyly of the extinct family Oviparosiphidae and its implications for inferring aphid evolution (Hemiptera, Sternorrhyncha)

**DOI:** 10.1371/journal.pone.0174791

**Published:** 2017-04-26

**Authors:** Dagmara Żyła, Agnieszka Homan, Piotr Wegierek

**Affiliations:** 1 Natural History Museum of Denmark, Biosystematics Section, Zoological Museum, Copenhagen, Denmark; 2 Department of Zoology, University of Silesia, Katowice, Poland; USDA Agricultural Research Service, UNITED STATES

## Abstract

Aphidoidea, the so-called "true aphids" are one of the most challenging groups in terms of solving the phylogenetic relationships. Morphology-based analyses were strongly affected by widespread homoplasy, while the molecular-based attempts struggled with the lack of sufficient phylogenetic signal. Despite significant improvements, the higher classification still remains unresolved and rather controversial. However, the use of the fossil record, one of the most valuable sources of information, was mainly limited to calibration of a phylogenetic tree, without a direct inclusion into the analysis. The extinct family Oviparosiphidae has long been considered as the common ancestor of all recent Aphidoidea and it was used as a calibration point in several analyses, but it has been never analyzed in a phylogenetic context. The family has been treated as a monophyletic group purely based on the simultaneous presence of two abdominal structures, ovipositor and siphunculi. However, it has been shown recently that at least one more extinct lineage, present at the same time, was characterized by the same features. For these reasons, we performed a maximum parsimony analysis using morphological data for extinct aphid taxa to prove the monophyly of Oviparosiphidae. Our analysis shows that the presumed ancestor lineage of recent aphids is a polyphyletic group. Our results support the hypothesis of an early Mesozoic rapid radiation of aphids, which led to several different lineages characterized by both ovipositor and siphunculi. The results indicate the necessity of examining the other extinct families, and shows that the diversity of aphids before the Cretaceous Terrestrial Revolution (KTR) was higher than expected. Even though there is not enough data to perform a formal analysis, fossils seem to suggest a significant impact of the KTR on aphid diversification. Additionally, we have made a redescription of two genera and description of a new species, *Vitimaphis subridens* sp. nov.

## Introduction

With ca. 5100 species worldwide [1], aphids (Hemiptera, Sternorrhyncha, Aphidomorpha sensu Heie & Wegierek [2]) represent one of the most biologically interesting groups. They have exceptionally complex life cycles, which include cyclical parthenogenesis and seasonal alternation between unrelated groups of host plants. They vary considerably in biological traits such as presence of many distinct, yet genetically identical forms of females during the life cycle, and the long-term mutualistic association with the endosymbiotic bacteria *Buchnera* [3]. Aphids are also a rare example of insects that are much more diverse in the temperate zone of the Northern Hemisphere than in the tropics or Southern Hemisphere [4, 5]. Similar with many members of Sternorrhyncha, they are small phloem-feeders and due to their remarkable ability to reproduce rapidly by viviparity, aphids are notorious agricultural pests. They damage plants not only by feeding on them, but also by transmitting almost 30% of all plant viruses [6]. All these make aphids an ideal group for solving various important basic and applied evolutionary questions. But, even though aphids attract much attention, their phylogeny that would be a framework for testing any evolutionary hypotheses, is hardly known. Naturally, higher classification of the group is not fully resolved as well.

### Aphid phylogeny—A review of published data

The first attempts to reconstruct aphid phylogeny based on morphology resulted in conflicting evolutionary scenarios [7, 8]. Although division of aphids into three main lineages—Aphidoidea with viviparous parthenogenetic females, and Adelgoidea and Phylloxeroidea with oviparous parthenogenetic females (classification follows [2]) were congruent among both studies, relationships within the Aphidoidea, which represent ca. 90% of recent aphid diversity, remained unresolved. A general impediment for phylogeny reconstruction and building natural classification of aphids is the paucity of morphological synapomorphies for higher-level lineages and difficulty in determining whether a certain feature is an ancestral (plesiomorphic) or derived (apomorphic) state [9]. First endeavours to apply DNA sequence data in aphid phylogenetics were based on mitochondrial genes and showed a striking lack of sufficient phylogenetic signal for taxon levels higher than tribe [3, 10]. These obstacles were thought to be a result of rapid radiations that occurred several times during aphid evolution, resulting in very weakly shared history experienced by individual clades [4]. On the contrary, very promising phylogenetic results were obtained by using DNA sequencies from their symbiotic bacteria *Buchnera* (e.g. [2]). As already known, aphids have mutualistic associations with *Buchnera aphidicola* and it was hypothesized that they have co-diversified in parallel since an original infection in the ancestor of modern aphids (e.g. [11]). However, some methodological issues raised doubts about the validity of the *Buchnera* phylogenies (for details see [12]), but in spite of potential defects, this hypothesis has been prevalent in literature (e.g. [2, 12]). Most recent phylogenetic studies on *Buchnera*, based on a wider taxon sampling and larger amount of genomic data, indicated incongruence with aphid phylogeny at higher taxonomic levels, which means that the parallel evolution of aphids and *Buchnera* needs to be re-considered at different taxonomic levels [13]. For these reasons, searching for a way to resolve the aphid phylogeny is still badly needed.

### Fossil record of the Mesozoic aphids

Nowadays we are witnessing an increased appreciation of the fossil record for reconstructing phylogeny of particular groups and testing macroevolutionary hypotheses (see e.g. [14, 15]). Fossils are a tremendous source of information regarding the tempo and mode of lineage diversification and trait evolution, thus the best way to use them is by analyzing data from living and fossil species together in a phylogenetic framework [15]. Despite the undoubted advantages, this approach also brings many challenges, especially for such a complex group as aphids, and it has been applied in a very limited way for studying aphid evolution, e.g. [16]. Although not very numerous, the Mesozoic fossils can provide very important data on the body structure at the early stages of aphid evolution, if they are carefully considered in phylogenetic context.

The oldest representative of a lineage presumably leading to recent aphids is *Leaphis prima* Shcherbakov, 2010, known from the early Anisian (Middle Triassic) of the Vosges, France [17]. Recently Szwedo et al. [18] described a new superfamily Lutevanaphidoidea from the Middle Permian of the Lodève Basin, France and assigned it to Aphidomorpha. However, in our opinion, the affiliation of this taxon to aphids should be further explored, e.g. because of the presence of a distinctly developed clavus on the fore wings. Most Triassic aphids are known from isolated wings, except the Dracaphididae from the superfamily Naibioidea, which were described from the Middle Triassic of China. The assignment of Naibioidea to aphids, however, remains controversial. Originally Shcherbakov [19] treated Naibioidea as a missing link between Aphidomorpha and their sister group Coccomorpha, but Hong et al. [20] and Heie & Wegierek [7, 21, 22] placed them as a superfamily within Aphidomorpha, refusing some of the characters presented by Shcherbakov [19]. Jurassic aphids are also scarce and only nine species were described [22, 23, 24]. The oldest, undoubted family for which body remains are known is Juraphididae, described from the Late Jurassic, and it is also the first family established from phylogenetic analysis [23] that was a good starting point for the subsequent attempts. A further 12 species are known from deposits of uncertain dating, estimated to the Late Jurassic or Early Cretaceous. From the Early Cretaceous aphid fossils have become much more numerous—more than 80 species, placed in 12 families [22, 25, 26, 27, 28]. A list of Mesozoic aphids is provided in Table 1. It is characteristic that the Late Cretaceous taxonomic composition of aphids differs significantly compared to the previous fauna, probably as a consequence of the Cretaceous Terrestrial Revolution (KTR), also referred to as the angiosperm revolution [29, 30].

**Table 1:** List of Mesozoic aphids.

Superfamily	Family	Subfamily	Genus	Species	Fossil sites
**Late Cretaceous**					
Palaeoaphidoidea	Palaeoaphididae		*Ambaraphis*	*A*. *costalis* Richards, 1966	Cedar Lake, Canada, Campanian [31]
				*A*. *kotejai* Kania & Wegierek, 2005	Alberta, Canada, Campanian [31]
			*Jersaphis*	*J*. *luzzii* Wegierek, 2000	New Jersey, USA, Turonian [31]
			*Longiradius*	*L*. *footitti* Heie, 2006	Alberta, Canada, Campanian [31]
			*Palaeoaphidiella*	*P*. *abdominalis* Heie, 1996	Alberta, Canada, Campanian [31]
			*Palaeoaphis*	*P*. *archimedia* Richards, 1966	Cedar Lake, Canada, Campanian [31]
				*P*. *armani* Wegierek, 1993	Obeshchayushchiy, Russia, Santonian/Campanian [32, 33]
				*P*. *incognita* Kononova, 1976	Taimyr Peninsula,Yantardakh, Russia, Santonian [31]
	Shaposhnikoviidae		*Shaposhnikovia*	*S*. *electri* Kononova, 1976	Taimyr Peninsula,Yantardakh, Russia, Santonian [31]
Tajmyraphidoidea	Tajmyraphididae		*Jantardakhia*	*J*. *electri* Kononova, 1975	Taimyr Peninsula,Yantardakh, Russia, Santonian [31]
			*Tajmyraphis*	*T*. *beckermigdisovae* Kononova, 1975	Taimyr Peninsula,Yantardakh, Russia, Santonian [31]
				*T*. *zherichini* Kononova, 1975	Taimyr Peninsula,Yantardakh, Russia, Santonian [31]
	Grassyaphididae		*Grassyaphis*	*G*. *pikei* Heie, 1996	Alberta, Canada, Campanian [31]
	Khatangaphididae		*Khatangaphis*	*K*. *sibirica* Kononova, 1975	Taimyr Peninsula,Romanikha, Russia, Santonian [31]
	Retinaphididae		*Retinaphis*	*R*. *glandulosa* Kononova, 1975	Taimyr Peninsula,Yantardakh, Russia, Santonian [31]
				*R*. *rasnitsyni* (Kononova, 1975)	Taimyr Peninsula,Yantardakh, Russia, Santonian [31]
Aphidoidea	Canadaphididae		*Alloambria*	*A*. *caudata* Richards, 1966	Cedar Lake, Canada, Campanian [31]
				*A*. *infelicis* Kania & Wegierek, 2005	Alberta, Canada, Campanian [31]
				*A*. *phoenicis* Wegierek, 1993	Obeshchayushchiy, Russia, Santonian/Campanian [32, 33]
			*Canadaphis*	*C*. *carpenteri* Essig, 1938	Cedar Lake, Canada, Campanian [31]
				*C*. *kovalevi* Wegierek, 1993	Obeshchayushchiy, Russia, Santonian/Campanian [32, 33]
				*C*. *mordvilkoi* Kononova, 1976	Taimyr Peninsula,Yantardakh, Russia, Santonian [31]
			*Pseudambria*	*P*. *longirostris* Richards, 1966	Cedar Lake, Canada Campanian [31]
	Cretamyzidae		*Cretamyzus*	*C*. *pikei* Heie, 1992	Alberta, Canada, Campanian [31]
	Parvaverrucosidae		*Parvaverrucosa*	*P*. *annulata* (Poinar & Brown, 2005)	Hukawng Valley, Myanmar, Albian/Cenomanian [31]
	Drepanosiphidae*		*Aniferella*	*A*. *bostoni* Richards, 1966	Alberta, Canada, Campanian [31]
				*A*. *sibirica* Kononova, 1977	Taimyr Peninsula,Yantardakh, Russia, Santonian [31]
			*Nordaphis*	*N*. *sukatchevae* Kononova, 1977	Taimyr Peninsula,Zhdanikha, Russia, Albian [31]
	Aphididae*		*Aphidocallis*	*A*. *caudatus* Kononova, 1977	Taimyr Peninsula,Yantardakh, Russia, Santonian [31]
Adelgoidea	Mesozoicaphididae		*Albertaphis*	*A*. *longirostris* Heie, 1992	Alberta, Canada, Campanian [31]
			*Calgariaphis*	*C*. *unguifera* Heie, 1992	Alberta, Canada, Campanian [31]
			*Campaniaphis*	*C*. *albertae* Heie, 1992	Alberta, Canada, Campanian [31]
			*Mesozoicaphis*	*M*. *canadensis* Heie, 1992	Alberta, Canada, Campanian [31]
				*M*. *electri* Heie, 1992	Alberta, Canada, Campanian [31]
				*M*. *parva* Heie, 1992	Alberta, Canada, Campanian [31]
				*M*. *tuberculata* Heie, 1992	Alberta Canada, Campanian [31]
	Elektraphididae		*Antonaphis*	*A*. *affinis* Kononova, 1977	Taimyr Peninsula,Yantardakh, Russia, Santonian [31]
				*A*. *brachycera* Kononova, 1977	Taimyr Peninsula,Yantardakh, Russia, Santonian [31]
			*Tajmyrella*	*T*. *cretacea* Kononova, 1976	Taimyr Peninsula,Yantardakh, Russia, Santonian [31]
**Early Cretaceous /Late Cretaceous boundary**					
Tajmyraphidoidea	Burmitaphididae		*Burmitaphis*	*B*. *prolatum* Poinar & Brown, 2005	Hukawng Valley, Myanmar, Albian/Cenomanian [31]
			*Caulinus*	*C*. *burmitis* Poinar & Brown, 2005	Hukawng Valley, Myanmar, Albian/Cenomanian [31]
**Early Cretaceous**					
Palaeoaphidoidea	Palaeoaphididae	Ellinaphidinae	11 genera [8, 25]	39 species [8, 25]	Baissa, Russia, Aptian [34, 35], Albian-Campanian [36]
			*Caudaphis*	*C*. *leptoneura* Zhang, Zhang, Hou & Ma, 1989	Laiyang, China, Aptian [34, 35]
				*C*. *minulissima* Zhang, Zhang, Hou & Ma, 1989	Laiyang, China, Aptian [34, 35]
				*C*. *spinalis* Zhang, Zhang, Hou & Ma, 1989	Laiyang, China, Aptian [34, 35]
	Szelegiewicziidae		*Brimaphis*	*B*. *abdita* Wegierek, 1989	Bon-Tsagan, Mongolia, Aptian [37]
				*B*. *certa* Wegierek, 1989	Baissa, Russia, Aptian [34, 35], Albian-Campanian [36]
				*B*. *similis* Wegierek, 1989	Baissa, Russia, Aptian [34, 35], Albian-Campanian [36]
			*Sepiaphis*	*S*. *versa* Wegierek, 1989	Bon-Tsagan, Mongolia, Aptian [37]
			*Szelegiewiczia*	*Sz*. *maculata* Shaposhnikov, 1985	Baissa, Russia, Aptian [34, 35], Albian-Campanian [36]
			*Tinaphis*	*T*. *laticubitus* Wegierek, 1989	Baissa, Russia, Aptian [34, 35], Albian-Campanian [36]
			*Xenoaphis*	*X*. *viticulata* Wegierek, 1989	Baissa Russia, Aptian [34, 35], Albian-Campanian [36]
	Rasnitsynaphididae		*Rasnitsynaphis*	*R*. *coniuncta* Homan & Wegierek, 2011	Baissa, Russia, Aptian [34, 35], Albian-Campanian [36]
				*R*. *ennearticulata* Homan & Wegierek, 2011	Baissa, Russia, Aptian [34, 35], Albian-Campanian [36]
				*R*. *quadrata* Homan & Wegierek, 2011	Baissa, Russia, Aptian [34, 35], Albian-Campanian [36]
	Juraphididae		*Aphaorus*	*A*. *curtipes* Wegierek, 1991	Khutel-Khara, Mongolia, Berriasian [37]
Tajmyraphidoidea	Burmitaphididae		*Alavesiaphis*	*A*. *margaritae* Peñalver & Wegierek, 2008	Peñacerrada, SpainAlbian [31]
	Khatangaphididae		*Khatangaphis*	*K*. *rohdendorfi* Kononova, 1975	Taimyr Peninsula,Kresty, Russia, Albian [31]
	Lebanaphididae		*Lebanaphis*	*L*. *minor* Heie, 2000	Levantinae amber, Lebanon, Barremian [31, 38]
			*Megarostrum*	*M*. *azari* Heie, 2000	Levantinae amber, Lebanon, Barremian [31, 38]
Aphidoidea	Oviparosiphidae		*Acanthotrichaphis*	*A*. *paulisensoriata* Shaposhnikov & Wegierek, 1989	Baissa, Russia, Aptian [34, 35], Albian-Campanian [36]
			*Archeoviparosiphum*	*A*. *baissense* Żyła, Homan, Franielczyk & Wegierek, 2015	Baissa, Russia, Aptian [34, 35], Albian-Campanian [36]
				*A*. *camtotropum* (Zhang, Zhang, Hou & Ma, 1989)	Laiyang, China, Aptian [34, 35]
				*A*. *latum* Hong & Wang, 1990	Laiyang, China, Aptian [34, 35]
				*A*. *malacum* (Zhang, Zhang, Hou & Ma, 1989)	Laiyang, China, Aptian [34, 35]
				*A*. *optimum* (Zhang, Zhang, Hou & Ma, 1989)	Laiyang, China, Aptian [34, 35]
				*A*. *tuanwangense* (Zhang, Zhang, Hou & Ma, 1989)	Laiyang, China, Aptian [34, 35]
			*Expansaphis*	*E*. *laticosta* Hong & Wang, 1990	Laiyang, China, Aptian [34, 35]
				*E*. *ovat*a Hong & Wang, 1990	Laiyang, China, Aptian [34, 35]
			*Dinaphis*	*D*. *multisensoriata* Shaposhnikov & Wegierek, 1989	Baissa, Russia, Aptian [34, 35], Albian-Campanian [36]
			*Oviparosiphum*	*O*. *jakovlevi* Shaposhnikov, 1979	Bon-Tsagan, Mongolia, Aptian [37]
			*Vitimaphis*	*V*. *rasnitsyni* Shaposhnikov & Wegierek, 1989	Baissa, Russia, Aptian [34, 35], Albian-Campanian [36]
				*V*. *subridens* sp. nov.	Baissa, Russia, Aptian [34, 35], Albian-Campanian [36]
			*Sinoviparosiphum*	*S*. *lini* Ren, 1995	Gaositai, China, Aptian [34, 35]
	Bajsaphididae		*Bajsaphis*	*B*. *abbreviate* Homan, Żyła & Wegierek, 2015	Baissa, Russia, Aptian [34, 35], Albian-Campanian [36]
				*B*. *cuspidate* Homan, Żyła & Wegierek, 2015	Baissa, Russia, Aptian [34, 35], Albian-Campanian [36]
				*B*. *eridmata* Homan, Żyła & Wegierek, 2015	Baissa, Russia, Aptian [34, 35], Albian-Campanian [36]
				*B*. *kononovae* Shaposhnikov, 1985	Baissa, Russia, Aptian [34, 35], Albian-Campanian [36]
				*B*. *pulchra* Homan, Żyła & Wegierek, 2015	Baissa, Russia, Aptian [34, 35], Albian-Campanian [36]
	Canadaphididae		*Nuuraphis*	*N*. *gemma* Wegierek, 1991	Bon-Tsagan, Mongolia, Aptian [37]
	Sinaphididae		*Sinaphidium*	*S*. *epichare* Zhang, Zhang, Hou & Ma, 1989	Laiyang, China, Aptian [34, 35]
			*Tartaraphis*	*T*. *peregrina* Zhang, Zhang, Hou & Ma, 1989	Laiyang, China, Aptian [34, 35]
	Hormaphididae*?		*Petiolaphioides*	*P*. *shandongensis* Hong & Wang, 1990	Laiyang, China, Aptian [34, 35]
			*Petiolaphis*	*P*. *laiyangensis* Hong & Wang, 1990	Laiyang, China, Aptian [34, 35]
	Drepanosiphidae*?		*Cretacallis*	*C*. *polysensoria* Shaposhnikov, 1979	Bon-Tsagan, Mongolia, Aptian [37]
	Thelaxidae*		*Gondvanoaphis*	*G*. *estephani* Wegierek & Grimaldi, 2010	Levantinae amber, Lebanon, Barremian [31, 38]
**Late Jurassic/Early Cretaceous boundary**					
Palaeoaphidoidea	Juraphididae		*Pterotella*	*P*. *formosa* Wegierek, 1991	Khotont, Mongolia, Jurassic/Cretaceous boundary [32, 37]
	Palaeoaphididae	Ellinaphidinae	*Secusellinaphis*	*S*. *khotontensis* Żyła & Wegierek, 2015	Khotont, Mongolia, Jurassic/Cretaceous boundary [32, 37]
			*Vetellinaphis*	*V*. *cracens* Żyła & Wegierek, 2015	Khotont, Mongolia, Jurassic/Cretaceous boundary [32, 37]
				*V*. *longalata* Żyła & Wegierek, 2015	Khotont, Mongolia, Jurassic/Cretaceous boundary [32, 37]
		Palaeoaphidinae	*Primpalaeoaphis*	*P*. *khotontensis* Żyła & Wegierek, 2013	Khotont, Mongolia, Jurassic/Cretaceous boundary [32, 37]
Naibioidea	Naibiidae		*Panirena*	*P*. *sukatshevae* Shcherbakov, 2007	Kempendyai, Russia, Jurassic/Cretaceous boundary [32]
				*P*. *tenella* Shcherbakov, 2007	Kempendyai, Russia, Jurassic/Cretaceous boundary [32]
Aphidoidea	Oviparosiphidae		*Khotontaphis*	*K*. *lachnoides* Shaposhnikov & Wegierek, 1989	Khotont, Mongolia, Jurassic/Cretaceous boundary [32, 37]
**Late Jurassic**					
Palaeoaphidoidea	Juraphididae		*Juraphis*	*J*. *crassipes* Shaposhnikov, 1979	Karatau, Kazakhstan, Oxfordian [32]
				*J*. *karataviensis* Żyła, Blagoderov & Wegierek, 2014	Karatau, Kazakhstan, Oxfordian [32]
			*Pterotella*	*P*. *shartegensis* Żyła, Blagoderov & Wegierek, 2014	Shar Teg, Mongolia, Late Jurassic [39]
Genaphidoidea	Genaphididae		*Genaphis*	*G*. *valdensis* (Brodie, 1845)	Vale of Wardour, England, Tithonian [40]
unknown	unknown		*Jurocallis*	*J*. *longipes* Shaposhnikov, 1979a:	Karatau, Kazakhstan, Oxfordian [32]
**Middle Jurassic**					
Aphidoidea	Oviparosiphidae		*Daoaphis*	*D*. *magnalata* Huang, Wegierek, Żyła & Nel, 2015	Daohugou, China, Callovian [41]
Naibioidea	Sinojuraphididae		*Sinojuraphis*	*S*. *ningchengensis* Huang & Nel, 2008	Daohugou, China, Callovian [41]
Palaeoaphidoidea	Szelegiewicziidae		*Tinaphis*	*T*. *sibirica* Wegierek, 1989	Kubekovo, Russia, Aalenian/Bathonian [32]
**Early Jurassic**					
Aphidoidea	Oviparosiphidae		*Grimmenaphis*	*G*. *magnifica* Ansorge, 1996	Grimmen, Germany, Toarcian [56]
**Middle Triassic**					
Triassoaphidoidea	Creaphididae	Leaphidinae	*Leaphis*	*L*. *prima* Shcherbakov, 2010	Vosges, France, Anisian [42]
Naibioidea	Dracaphididae		*Dracaphis*	*D*. *angustata* Hong, Zhang, Guo & Heie, 2009	Hejiafang Village, China, Ladinian [20]
unknown	unknown		*Dubiaphis*	*D*. *curvata* Brauckmann & Schlüter, 1993	Hammelburg, Germany, Anisian [43]
**Late Triassic and Late/Middle Triassic boundary**					
Triassoaphidoidea	Triassoaphididae		*Triassoaphis*	*T*. *cubitus* Evans, 1956	Mt. Crosby, Australia, Norian [44]
	Creaphididae	Creaphidinae	*Creaphis*	*C*. *theodora* Shcherbakov & Wegierek, 1991	Dzhailoucho, Kyrgyzstan, Ladinian-Carnian [45]
Naibioidea	Naibiidae		*Coccavus*	*C*. *supercubitus* Shcherbakov, 2007	Dzhailoucho, Kyrgyzstan, Ladinian-Carnian [45]

Colors correspond to the analyzed families.

* Extant families are marked with asterisk.

For the exact list of the Early Cretaceous Ellinaphidinae see Heie & Wegierek [8], and Kania & Wegierek [25].

One of the oldest known extinct families of aphids is Oviparosiphidae, described from the Middle Jurassic to the late Early Cretaceous [22, 46]. The family currently consists of only 15 species classified into 10 genera, but they are highly diverse morphologically [22, 24, 47]. It is the first known group with a special structure on the dorsal part of the abdomen, the so-called siphunculi. However, they still maintained the ovipositor. Such combination does not occur in any recent family, and has also been considered unique among extinct groups. For a long time, only one other family with the same combination of features has been identified, the Late Cretaceous Canadaphididae. And traditionally, almost each aphid with the simultaneous presence of ovipositor and siphunculi that was found in sediments up to the end of the Early Cretaceous was classified to Oviparosiphidae, while those from the Upper Cretaceous deposits to the Canadaphididae. Recently, however, a newly described family Bajsaphididae (Early Cretaceous) showed that this condition has convergently evolved in more than one group [27], which led us to question whether Oviparosiphidae really formed a natural group.

Since the family is considered to be the stem group of all recent Aphidoidea, resolving this uncertainty is critically important for assessing important morphological character polarity and investigating patterns that determined early steps of aphid evolution. In this paper, we rigorously test the monophyly of Oviparosiphidae by means of phylogenetic analysis using maximum parsimony (MP) method, which provides a step in the desired direction. Additionally, we have made a redescription of the type species of two genera and a description of new species, which provide more morphological details.

## Material and methods

### Specimen depositories

All specimens examined here are from the Laboratory of Arthropods, Institute of Palaeontology, Russian Academy of Science, Moscow (PIN) collection. Imprints were collected from two localities: Khotont (Upper Jurassic/Lower Cretaceous boundary, Mongolia) and Baissa (Berriasian, Lower Cretaceous, Russia) [48].

### Nomenclatural acts

The electronic edition of this article conforms to the requirements of the amended International Code of Zoological Nomenclature, and hence the new names contained herein are available under that Code from the electronic edition of this article. This published work and the nomenclatural acts it contains have been registered in ZooBank, the online registration system for the ICZN. The ZooBank LSIDs (Life Science Identifiers) can be resolved and the associated information viewed through any standard web browser by appending the LSID to the prefix “http://zoobank.org/”. The LSID for this publication is: urn:lsid:zoobank.org:pub: 6E9E7CA1-CF53-41C9-8914-8013089AA1B5. The electronic edition of this work was published in a journal with an ISSN, and has been archived and is available from the following digital repositories: PubMed Central, LOCKSS.

### Examination of fossils

Fossil specimens are preserved in the form of two imprints—the ‘reverse’ and the ‘obverse’. Each specimen of the PIN collection has a unique number, i.e. the collection number, representing the collection event or locality, and the serial number of the specimen, such as 4307/44±. The techniques used for studying the imprints follow those of Rasnitsyn [49]. Specimens were photographed using a Nikon SMZ1500 stereoscopic microscope, a polarized Nikon Eclipse-E600 light microscope and a Philips XL 30 TMP ESEM scanning electron microscope (using the secondary electron detector). Owing to the oxidation of iron on the surface of the rocks, the material from Khotont had to be studied without alcohol, but using the polarized light microscope. For *Vitimaphis* specimens a digital Tablet was applied to make the drawings on the photographic layer in Adobe Photoshop CS3, while *Khotonaphis* drawings were made from photographs and digitally inked in Adobe Illustrator C6. The figures are based on the combined drawings of the reverse and obverse imprints, but photographs represent only one imprint. All measurements are given in mm.

### Phylogenetic analysis

The morphological data matrix was constructed with Mesquite 3.03 [50]. It includes 39 characters (numbered 1–39) scored for 14 taxa. Due to the lack of access to the material, and their poor description and illustration, the following taxa of Oviparosiphidae are not included in the analysis: *Archeoviparosiphum camptotropum* (Zhang, Zhang, Hou & Ma, 1989), *A*. *latum* (Hong & Wang, 1990), *A*. *malacum* (Zhang, Zhang, Hou & Ma, 1989), *A*. *opimum* (Zhang, Zhang, Hou & Ma, 1989), *A*. *tuanwangense* (Zhang, Zhang, Hou & Ma, 1989), *Expansaphis laticosta* Hong & Wang, 1990, *E*. *ovata* Hong & Wang, 1990, *Grimmenaphis magnifica* Ansorge, 1996, and *Sinoviparosiphum lini* Ren, 1995 (for discussion of their status see [47]). Unknown character states were coded with “?”, inapplicable states with “–”. The character matrix is provided as S1 File in Nexus format. The maximum parsimony analyses were conducted in TNT 1.1 [51] using the “traditional search” option to find most parsimonious trees (MPTs) under the following parameters: memory set to hold 100000 trees; tree bisection—reconnection (TBR) branch-swapping algorithm with 100 replications saving 100 trees per replicate; zero-length branches collapse after the search. All character states were treated as unordered and equally weighted. Moreover, we also performed the separate analyses under implied weights of characters [52]. Value of the constant of concavity *k* determines how strongly homoplasious characters are down-weighted, and thus comparing the results obtained with different values allow to assess a stability of results [52]. The *k*-values ranged from 1, which represents a stronger down-weighting of the homoplasious characters, to 10, and represents a milder down-weighting. Bremer supports were calculated using the TNT Bremer function, using suboptimal trees up to 20 steps longer. Character mapping was made in WinClada v1.00.08 [53] using fast optimization (ACCTRAN), while trees were annotated in Adobe Illustrator C6. Genus *Juraphis* (Juraphididae) was used as the outgroup, as presumably the most phylogenetically remote taxon to the Oviparosiphidae.

### List of characters

The character list for the dataset is presented below:

1. Body: (0) thick (e.g. *Khotonaphis lachnoides*); (1) slender (all Canadaphididae)
2. Epicranial suture: (0) present; (1) absent
3. Lateral sutures: (0) present; (1) absent
4. Lateral sutures: (0) connected; (1) not connected
5. Rostrum: (0) very short, reaching fore coxae; (1) longer, reaching hind coxae
6. Antennae: (0) bead-like; (1) bristly (all Canadaphididae)
7. Antennal segments: (0) more than 6; (1) 6 or less
8. Length of antennae: (0) less or equal to 1/3 of body length; (1) longer than 1/3 of body length
9. Length of antennae: (0) equal or shorter than fore tibia; (1) longer than fore tibia
10. Length of antennae: (0) equal or shorter than hind tibia; (1) longer than hind tibia
11. Segment III of antennae: (0) short, 1/2 as long as subsequent segments; (1) long, almost equal to subsequent segments
12. Segment III of antennae: (0) less or equal to 3.5× longer than wide; (1) more than 3.5 but less than 4.5× longer than wide; (2) more than 8× longer than wide
13. Antennal segments from III to penultimate: (0) the same length; (1) various lengths
14. Last antennal segment: (0) shorter or equal to previous segment; (1) longer than previous segment
15. Last antennal segment narrowed: (0) absent; (1) present (e.g. *Vitimaphis rasnitsyni*)
16. Processus terminalis: (0) absent; (1) weakly developed
17. Primary rhinaria: (0) absent; (1) present
18. Hind tibia: (0) short, at most 1/3 of body length; (1) longer than 1/3 of body length
19. Hind tarsus: (0) short, more than 1/4 of hind tibia length; (1) long, less than 1/4 of hind tibia length
20. Fore wings: (0) longer than body; (1) equal to or shorter than body
21. Common stem of cubital veins: (0) present; (1) absent
22. Cubital veins: (0) both present; (1) only CuA_1_ present (an autapomorphy of *Pseudambria*)
23. Cubital veins: (0) bases reach the main vein at the same point, but veins are separated throughout their course; (1) bases separate but close (e.g. *Khotonaphis lachnoides*); (2) bases separate and far apart (e.g. *Vitimaphis rasnitsyni*); (3) CuA_1_ does not reach main vein
24. Cubital veins: (0) CuA_1_ at most 2.5× longer than CuA_2_; (1) CuA_1_ at least 3× longer than CuA_2_
25. Vein CuA_1_: (0) arcuate; (1) sinuate (all Canadaphididae)
26. Base of vein M: (0) closer to base of pterostigma (1) mid-distance between base of pterostigma and CuA_1_, or closer to base of CuA_1_
27. Ramification of M: (0) behind base of Rs; (1) before base of Rs; (2) at level of Rs base
28. Base of vein Rs: (0) closer to proximal part of pterostigma; (1) in mid-part of pterostigma; (2) closer to distal part of pterostigma
29. Vein Rs: (0) slightly curved (its apical part is located at the height of the lowest point of its course); (1) strongly curved (its apical part is located higher or lower than the lowest point of its course)
30. Vein Rs: (0) runs close to pterostigma (arising from pterostigma at an angle less than or equal to 45°); (1) runs far from pterostigma (arising from pterostigma at an angle greater than 45°)
31. Pterostigma, ending: (0) rounded; (1) pointed
32. Pterostigma, length: (0) short, at most 3.5× longer than wide; (1) long, at least 4× longer than wide
33. Ovipositor: (0) well developed (Bajsaphididae); (1) slightly reduced (*Oviparosiphum*); (2) rudimentary
34. Siphunculi on the abdomen: (0) absent; (1) present
35. Siphunculi on the abdomen, state of development: (0) porous; (1) conical
36. Cauda: (0) absent; (1) present
37. Anal plate: (0) not bilobed; (1) bilobed
38. Anal plate: (0) absent or rudimentary; (1) well developed (e.g. *Vitimaphis rasnitsyni*)
39. Setae on abdomen: (0) absent; (1) present (e.g. *Khotonaphis lachnoides*)

## Results

### Systematic palaeontology

Infraorder Aphidomorpha Becker-Migdisova & Aizenberg, 1962

Superfamily Aphidoidea Latreille, 1802

Family Oviparosiphidae Shaposhnikov, 1979

#### Genus *Khotonaphis* Shaposhnikov & Wegierek, 1989

Type species *Khotonaphis lachnoides* Shaposhnikov & Wegierek, 1989 by original designation and monotypy.

Emended diagnosis. Dorsal side of abdomen with rows of small tubercles.

*Khotonaphis lachnoides* Shaposhnikov & Wegierek

Figs 1 and 2.

**Fig 1:**
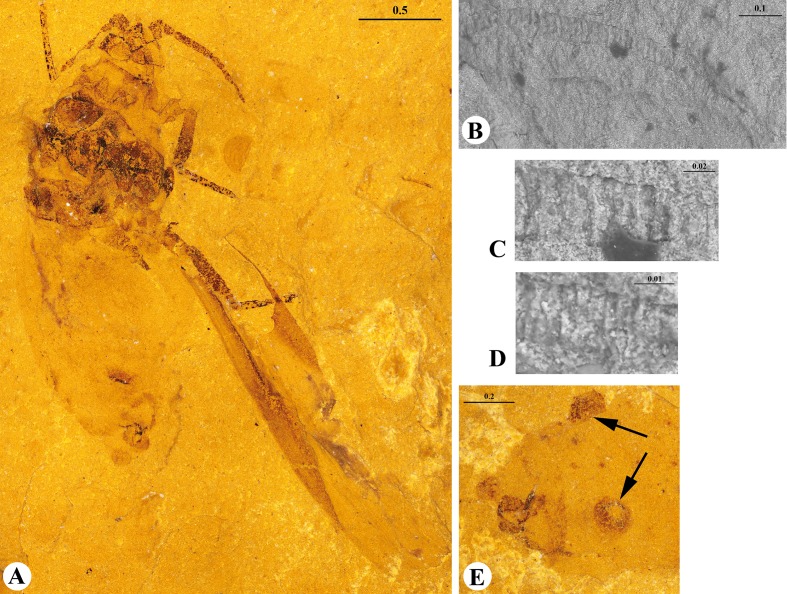
*Khotontaphis lachnoides* Shaposhnikov & Wegierek, 1989. A. 4307/150±, additional material, habitus (dorsal view); B. Additional material, scanning electron micrograph of right antenna; C. Additional material, scanning electron micrograph of fragment of III antennal segment; D. Additional material, scanning electron micrograph of fragment of VI antennal segment; E. 4307/194±, holotype, siphunculi (arrows). Scale bars in mm.

**Fig 2:**
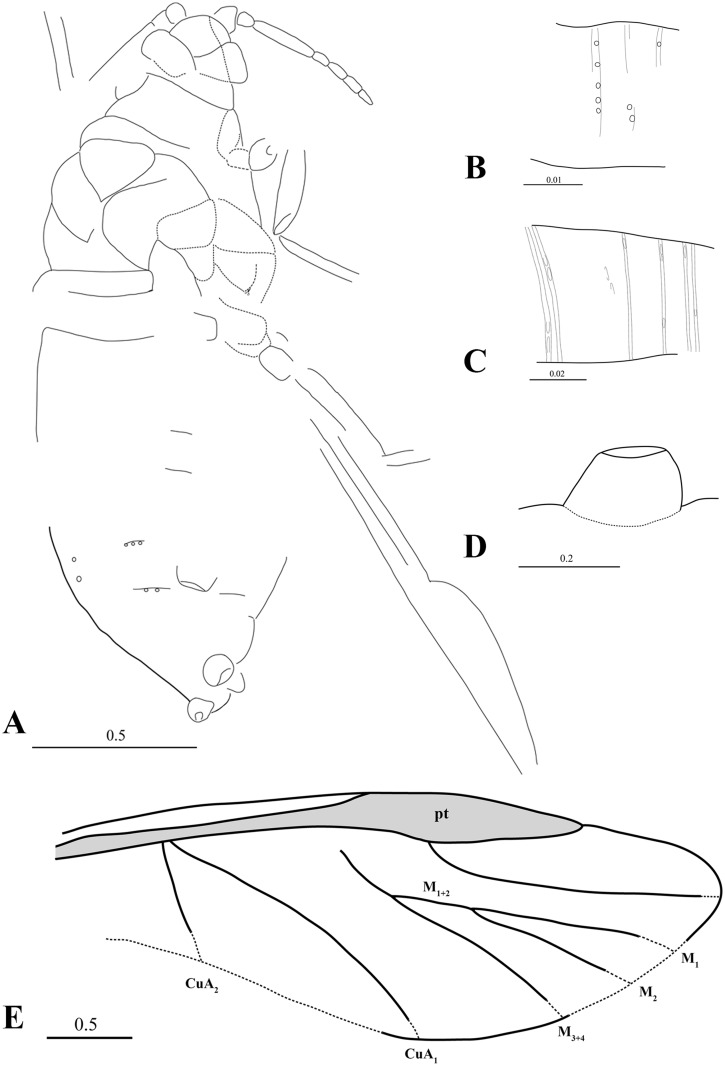
*Khotontaphis lachnoides* Shaposhnikov & Wegierek, 1989, line drawings. A. 4307/150±, additional material, habitus (dorsal view); B. Additional material, fragment of VI antennal segment; C. Additional material, fragment of III antennal segment; D. 4307/194±, holotype, siphunculus; E. Holotype, reconstruction of fore wing. Scale bars in mm.

Types. Holotype: 4307/194±, additional material: 4307/150±, 4307/57.2±.

Type locality and horizon. Khotont (Hotont), 6 km W Khotont Sum, Arhangai, Mongolia; Upper Jurassic or Lower Cretaceous.

Emended diagnosis. As for genus.

Redescription.

Body length 2.30–3.02 (Figs 1A and 2A). Head approximately 1/2 as long (0.23) as wide (0.48) with anterior margin rounded. Lateral sutures present, connected in middle of the epicranium. Diameter of ocelli 0.04. Antennae 7-segmented (0.89) (Fig 1B), 1/4 of body length, and nearly as long as hind tibiae. Antennal segment III (0.31) 5.5× as long as wide, somewhat shorter than other flagellar segments. Antennal segments IV and V of equal length (0.10), 2.5× as long as wide. Antennal segments VI and VII (0.09, 0.08) 3× as long as wide, tapering towards apex. Rhinaria present on all flagellar segments; elongated and ellipsoidal on segments III, IV and V (Figs 1C and 2C); tiny and rounded on segments VI and VII (Figs 1D and 2B). Pronotum 1/2 as long (0.12–0.24) as wide (0.39–0.52). Length of praescutum 0.16–0.23, width 0.29–0.32. Femur stouter than tibia in all legs. Lengths of fore, middle and hind femur: 0.55, 0.51 and 0.50–0.59, respectively. Tibia 1/4 of body length. Forewings longer than body (3.10–3.59). Distance between wing base and end of pterostigma 2.36–2.97. Veins CuA_1_ and CuA_2_ separating from main vein almost at the same point (Fig 2E). CuA_1_ (1.85) 3× as long as CuA_2_ (0.67). CuA_1_ leaving main vein at 35° angle, whereas CuA_2_ at 80° angle. Vein M with 3 branches. Vein M bifurcation narrow (25°), before Rs base. M common stem (0.32) as long as M_1+2_, and 3× shorter than M_3+4_ ((1.06). Vein Rs slightly curved, separating from pterostigma near its base at 30° angle and running close to it. Pterostigma pointed, long and narrow, about 5× as long (1.03–1.10) as wide (0.21–0.26). Siphunculi at base 2× as wide (0.21) as high (0.11) (Figs 1E and 2D). Diameter of siphunculi aperture 0.09. On the dorsal side of abdomen two longitudinal or several transverse rows of tubercles, scattered between siphunculi.

#### Genus *Vitimaphis* Shaposhnikov & Wegierek, 1989

Type species *Vitimaphis rasnitsyni* Shaposhnikov & Wegierek by original designation and monotypy.

Emended diagnosis. Antennae with 7 segments, short and thick, shorter than length of tibia. Segment III 2.5–3.5× as long as wide. Secondary rhinaria arranged in transverse rows. Veins CuA_1_ and CuA_2_ separated at bases. Vein CuA_1_ at most 1.8× longer that CuA_2_. Vein Rs arising from middle of pterostigma. Common stem of vein M arising from base of pterostigma. Anal plate large, triangular.

Redescription.

Body thick. Lateral sutures on head present, connected in middle of the epicranium. Anterior margin of head rounded. Ocelli large. Antennae at most 1/3 of body length. Last antennal segment narrowing from 1/2 of its length and blunt. All flagellar segments covered with transverse rib-like structures. Rhinaria ellipsoidal, arranged in dense rows; rows rarely connected. Anterior margin of mesoscutellum covex. Anterior margin of mesothoracic sternite straight, with large lateral arms and corners at level of transverse suture. Hind tibia shorter than 1/2 of body length. Pterostigma short and thick, 3× as long as wide, pointed. Vein Rs weakly curved, nearly straight. Vein M with 3 widely separated branches. Vein M bifurcating into M_1+2_ and M_3+4_ at level of Rs base. M common stem longer than vein M_1+2_. Hindwings with two straight cubital veins lying wide apart. Abdomen weakly sclerotized. Siphunculi slightly conical.

*Vitimaphis rasnitsyni* Shaposhnikov & Wegierek, 1989

Figs 2 and 3

**Fig 3:**
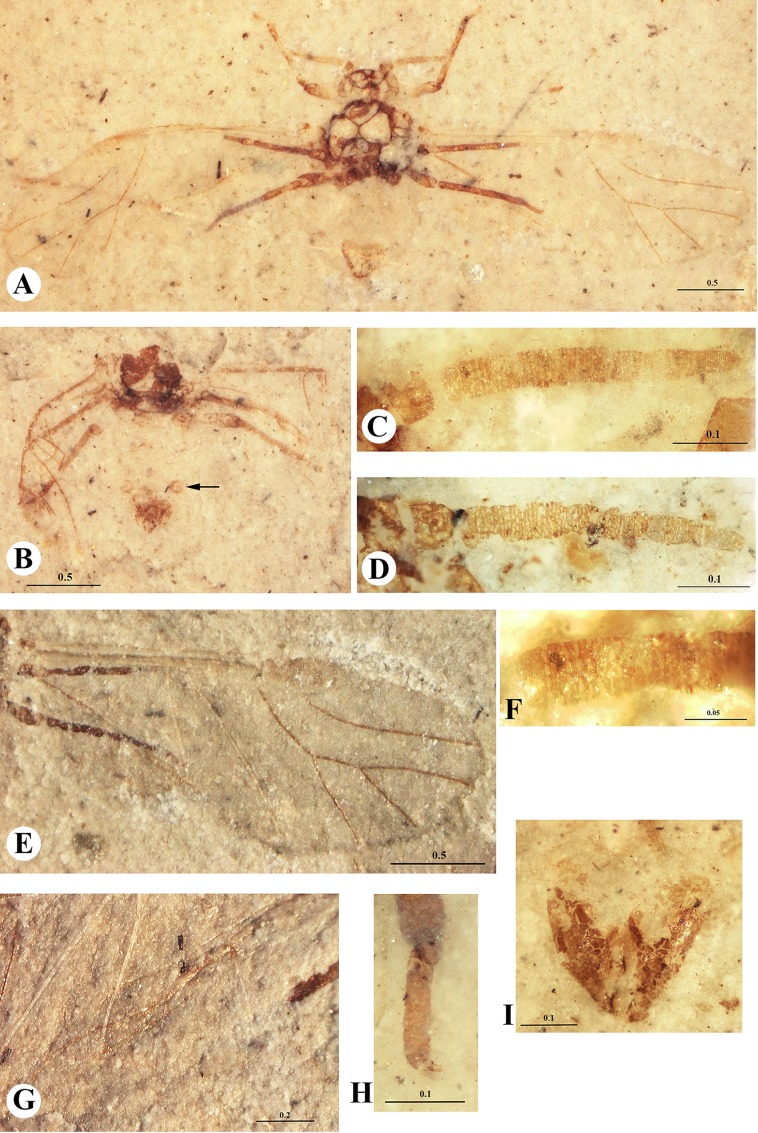
*Vitimaphis rasnitsyni* Shaposhnikov & Wegierek, 1989. A. 3064/2235, holotype, habitus (ventral view); B. 3064/5026(5062), paratype, habitus (dorsal view, siphunculus pointed by arrow); C. Holotype, right antenna; D. 3064/2092(2103), additional material, left antenna; E. Holotype, fore wing; F. Holotype, secondary rhinaria on III and IV antennal segments; G. Holotype, hind wing; H. Holotype, hind tarsus; I. 3064/5026(5062), paratype, anal plate. Scale bars in mm.

Types. Holotype: 3064/2235, paratype: 3064/2236; 3064/5026(5062), additional material: 3064/2092(2103).

Type locality and horizon. Baissa, Transbaikalia (Russia), Lower Cretaceous.

Emended diagnosis. Antennal segment III 2.5–3× as long as wide. Antennal segments IV, V, VI square. Secondary rhinaria large.

Redescription.

**Fig 4:**
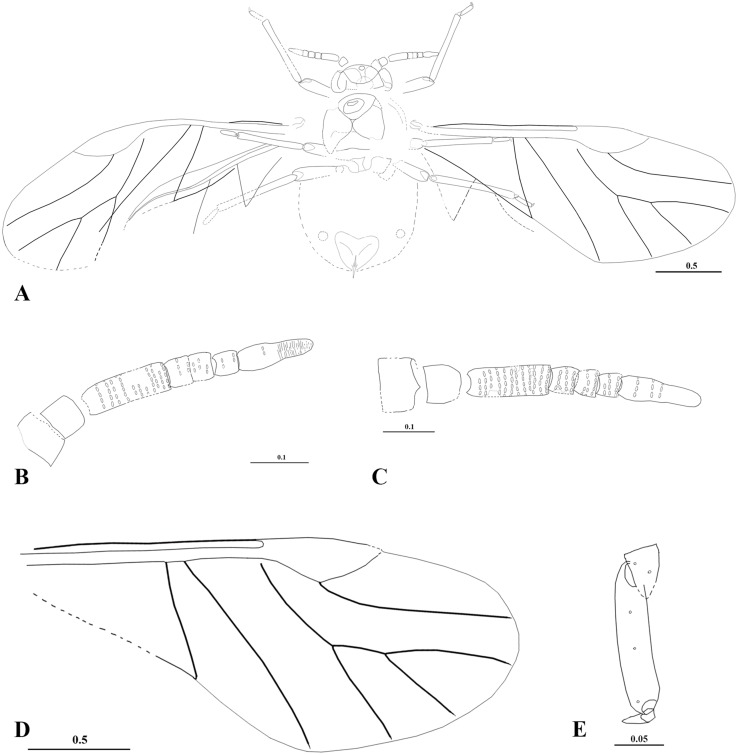
*Vitimaphis rasnitsyni* Shaposhnikov & Wegierek, 1989, line drawings. A. 3064/2235, holotype, habitus (ventral view); B. Holotype, right antenna; C. 3064/2092(2103), additional material, left antenna; D. Holotype, fore wing; E. Holotype, hind tarsus. Scale bars in mm.

Body length 1.5–1.6 (Figs 3A and 4A). Head width 0.32–0.34; length 0.16–0.19. Lateral sutures connected at 3/4 of head length. Antennae length 0.46–0.54. Antennal segments I and II 0.05–0.06; segment III 0.14–0.15; segments IV, V and VI 0.04. Antennal segment (VII 0.13–0.14) longer than cumulative length of segments IV-VI and nearly as long as segment III (Figs 3C, 3D, 4B and 4C). No more than 5 rhinaria in each of 12 rows on antennal segment III (Fig 3F). Other flagellar segments with at most 3 rows of rhinaria. Mesothoracic sternite width 0.47; height 0.24. Length of fore tibia 0.55–0.57; tarsal segment I 0.03–0.04; tarsal segment II 0.12–0.13. Length of middle tibia 0.49–0.56; tarsal segment I 0.03–0.04, tarsal segment II 0.12–0.13. Length of hind femur 0.3–0.45; tibia 0.6–0.71; tarsal segment I 0.04–0.05; tarsal segment II 0.15–0.16, 3.5× as long as tarsal segment I (Figs 3H and 4E). Forewing length 2.6; width 1.1 (Figs 3E and 4D). Distance between wing base and end of pterostigma 1.7–1.8. Distance between veins CuA_1_ and CuA_2_ 0.08–0.1. Hindwing length 1.6 (Fig 3G). Subgenital plate transverse (Fig 3B). Ovipositor rudimentary (Fig 3I).

*Vitimaphis subridens* sp. nov. urn:lsid:zoobank.org:act:9190D584-B00B-4D1F-82FF-07978FB2B219

Figs 5 and 6

**Fig 5:**
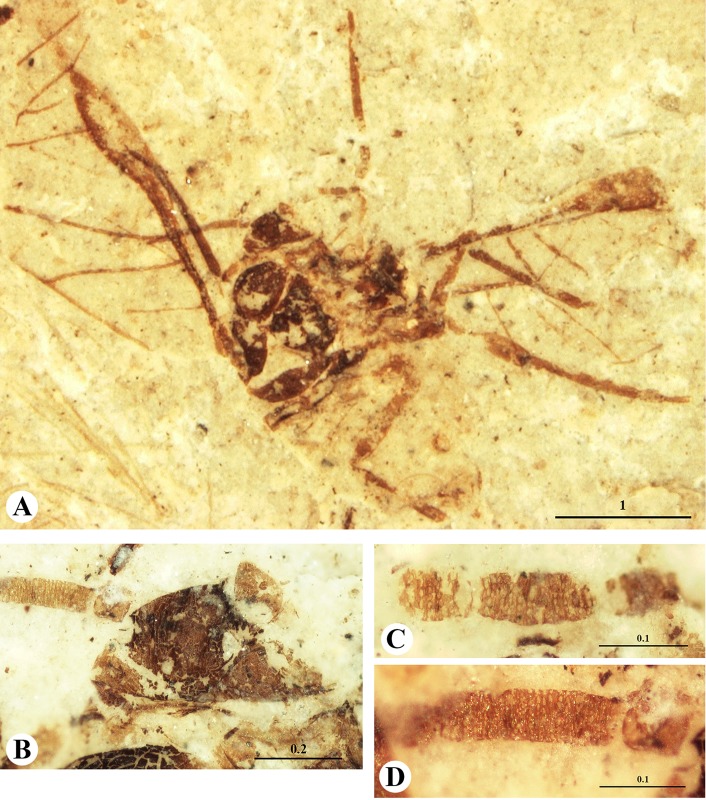
*Vitimaphis subridens* sp. nov. A. 3064/5000, holotype, habitus (dorsal view); B. Holotype, head; C. Holotype, fragment of right antenna; D. Holotype, fragment of left antenna. Scale bars in mm.

**Fig 6:**
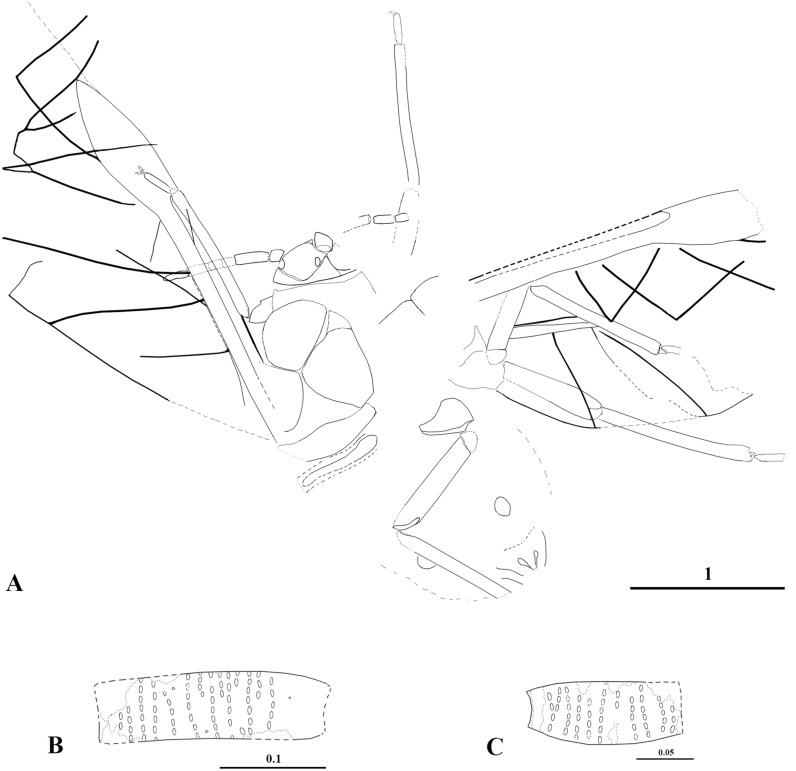
*Vitimaphis subridens* sp. nov., line drawings. A. 3064/5000, holotype, habitus (dorsal view); B. Holotype, secondary rhinaria on III antennal segment; C. Holotype, secondary rhinaria on IV antennal segment. Scale bars in mm.

Types. Holotype: 3064/5000+.

Type locality and horizon. Baissa, Transbaikalia (Russia), Lower Cretaceous.

Etymology. From the Latin *subridens*–smiling.

Diagnosis. Antennal segment III 3.5× as long as wide. Segments IV, V and VI rectangular. Secondary rhinaria small.

Description.

Body length 2.7 (Figs 5A and 6A). Head width 0.43; length 0.26. Lateral sutures connected at 4/5 of head length (Fig 5B). Distance between ocelli 0.2. Antennae length 0.86. Antennal segment I 0.07, segment II 0.08. Antennal segment III 0.22, 1/2 of cumulative length of segments IV–VII. Antennal segment IV 0.14, more than 1/2 as long as segment III. No more than 7 rhinaria in each of 15 rows on segment III (Figs 5D and 6B). Segment IV with 11 rows of rhinaria (Figs 5C and 6C). Mesothoracic sternite length 0.5. Length of fore and middle tibia 0.96. Segment II of fore tarsus 0.21. Hind coxa length 0.2; femur 0.75; tibia 1.14. Forewing length 3.6. Distance between wing base and end of pterostigma 2.7. Distance between veins CuA_1_ and CuA_2_ 0.2.

*Vitimaphis* incertae sedis:

3064/2236; 4210/2516(2518a+); 4210/2546+; 4210/4477.

### Phylogenetic analysis

**Fig 7:**
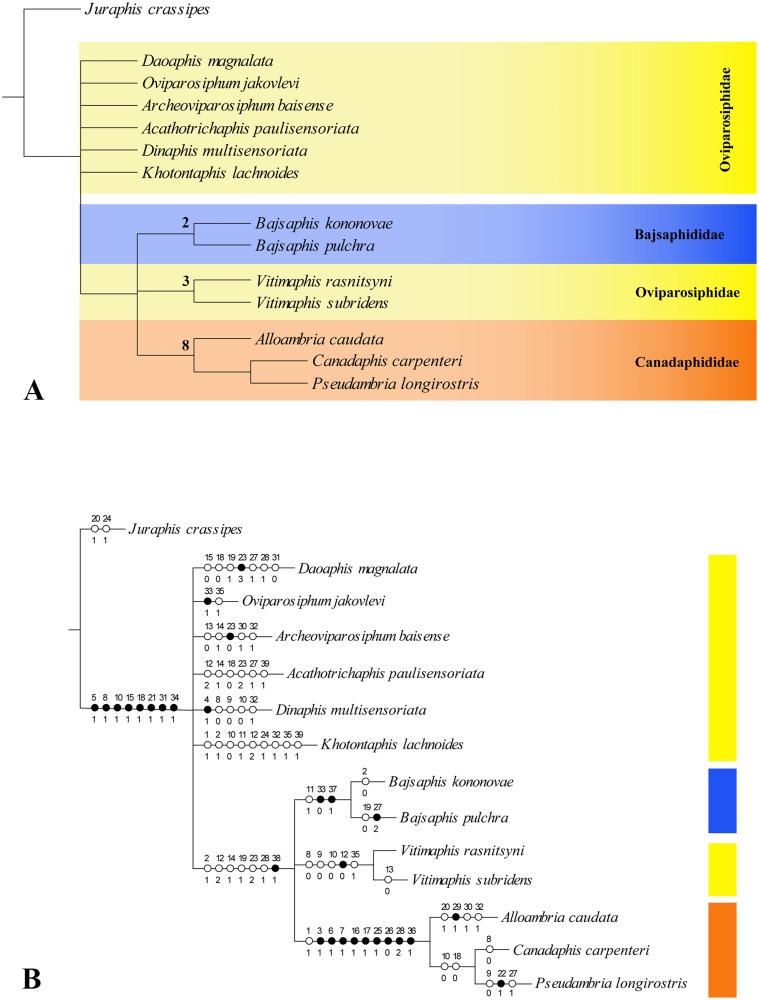
Results of Maximum Parsimony (MP) analyses. A. Strict consensus of the three most parsimonious trees with Bremer support (indicated by numbers at nodes); B. Strict consensus of the three most parsimonious trees with characters mapped. Circles with numbers along branches indicate synapomorphies (autapomorphies for terminal branches): black—unique changes; white—homoplasious changes; character numbers above circles, character state numbers below circles.

**Fig 8:**
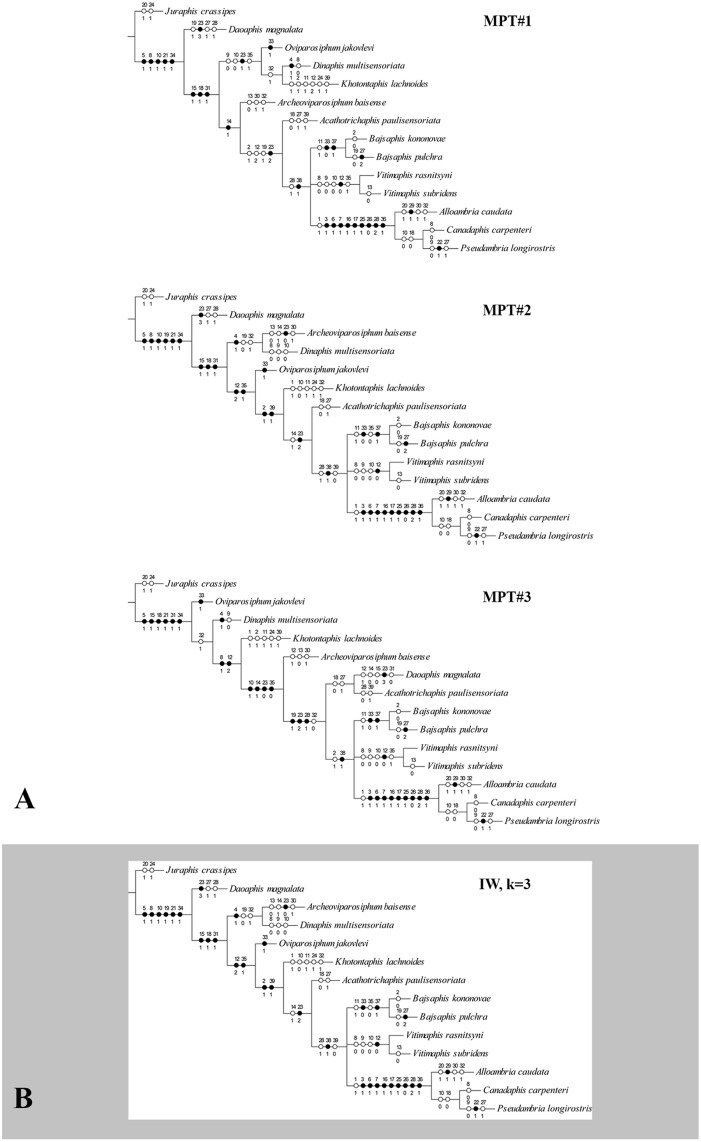
Results of Maximum Parsimony (MP) analyses. A. MPTs with characters mapped. Circles with numbers along branches indicate synapomorphies (autapomorphies for terminal branches): black—unique changes; white—homoplasious changes; character numbers above circles, character state numbers below circles. B. Result of MP analysis under implied weights of characters, k = 3. Circles and numbers as above.

The analysis under equal weights (EW) resulted in 3 equally most parsimonious trees (MPTs) with 76 steps, consistency index (CI) = 0.59, retention index (RI) = 0.63. The strict consensus tree was unresolved for Oviparosiphidae (Fig 7A and 7B). The individual MPTs (so-called here MPT#1, MPT#2, and MPT#3) differ substantially in the position of the Oviparosiphidae taxa, except *Vitimaphis*, which was recovered at the same position in every MPT (Fig 8A). Re-analysis under implied weights (IW) and at each *k*-value resulted in a single parsimonious tree in each run (tree obtained at *k* = 3 is shown in Fig 8B). All trees obtained with implied weighting did not differ from each other and they were consistent with the unweighted MPT#2. Overall, the main difference in the topology of all trees obtained under different searching strategies was a highly unstable position of most Oviparosiphidae taxa, which is clearly shown on the strict consensus tree, that resulted from EW searching (Fig 7). However, the families Canadaphididae and Bajsaphididae were recovered as monophyletic in all analyses. Our discussion is mostly based on the EW strict consensus tree, otherwise it is clearly stated which result is discussed.

## Discussion

### Monophyly of Oviparosiphidae + Bajsaphididae + Canadaphididae

Based on the simultaneous presence of ovipositor and siphunculi in Oviparosiphidae, Bajsaphididae and Canadaphididae, some authors hypothesized, e.g. [8, 27] that they constitute one lineage. Our analysis supports this view by recovering the Oviparosiphidae + Bajsaphididae + Canadaphididae group as monophyletic. Because it has the oldest known representative, the family Oviparosiphidae was treated as the ancestor of this lineage. The family is known from the relatively short time span from Middle Jurassic to Early Cretaceous, and its stratigraphic range partly overlaps with Bajsaphididae known from the Early Cretaceous. In subsequent geological epochs, the presence of these families has not been confirmed. One more family has been described from the Early Cretaceous of China, the Sinaphididae, but its systematic assignment is disputable. As postulated by Wegierek [in 54], Sinaphididae should be merged into one family with Oviparosiphidae, judging from their great similarity to each other. However, since the material is not available for revision, we did not include Sinaphididae in our analysis and did not make any taxonomic change. The Canadaphididae has been regarded as the successors of Oviparosiphidae, as they are mostly known from the Late Cretaceous. Only one species has been described from the Early Cretaceous, *Nuuraphis gemma* Wegierek, 1991, but the preservation is poor and even in the original description the author noted that its assignment is arbitrary and part of the diagnostic features was invisible [55].

### Monophyly of Bajsaphidae and Canadaphididae

Both outgroups were recovered as monophyletic families, supported by several synapomorphies. Bajsaphididae are known from the Baissa deposit (Russia) where its first known representative was originally described without assignment to any family. Even though recently some new species were described in Bajsaphidae, they were all placed in *Bajsaphis* leaving the family monogeneric [27]. Monophyly of *Bajsaphidae* is supported here by two synapomorphies (33, 37) that are consistent with the diagnostic features of the family. Canadaphididae, the second outgroup in our analysis, was also well supported as a monophylum based on 9 synapomorphies (3, 6, 7, 16, 17, 25, 26, 28, 36). This is not surprising because morphologically they differ significantly from Oviparosiphidae and Bajsaphididae, but at the same time still have some common features. They are considered descendants of the two latter families, but here we can neither confirm nor reject this hypothesis since the relationships within Oviparosiphidae and between Oviparosiphidae and Bajsaphididae are not resolved.

### Polyphyly of Oviparosiphidae

The analysis revealed that the family Oviparosiphidae does not constitute a monophylum. Oviparosiphid taxa changed their position on different trees, and only the position of the genus *Vitimaphis* is more stable and it is nested within the Bajsaphididae + Canadaphididae group. The MPT#1 and MPT#2 (Fig 8A) showed the genus *Daoaphis* Huang, Wegierek, Żyła & Nel, 2015 as a sister group for all other taxa. The same result was also revealed under the IW searching strategy. So far, this genus, described from the Daohugou deposit (Middle Jurassic of China) [24], is the oldest known unambiguous Oviparosiphid taxon because taxonomic placement of the older *Grimmenaphis magnifica* Ansorge, 1996, known from an isolated wing from the Upper Liassic (Lower Jurassic) Grimmen deposit (Germany) [56], is questionable. Under the IW searching strategy, the genus *Acathotrichaphis* Shaposhnikov & Wegierek, 1989 was found as a sister group to the Bajsaphididae + *Vitimaphis* + Canadaphididae group (Fig 8B). In the original description, the authors were not sure whether this genus has an ovipositor, and they stated that its assignment to the Oviparosiphidae is arbitrary [57]. However, judging from their drawings for *Acathotrichaphis* and personal observation (PW), we can see the ovipositor-like structure at the end of the abdomen. On MPT#3 these two genera (*Daoaphis* and *Acathotrichaphis*) form a clade sister to Bajsaphididae + *Vitimaphis* + Canadaphididae (Fig 8A), which may indicate that they actually somewhat differ from other taxa. On the same tree, the genus *Oviparosiphum* Shaposhnikov, 1979 is recovered as a sister to all other taxa that seems to be highly unlikely. For a long time, the genus *Oviparosiphum* consisted of seven species, but new material allowed a detailed revision resulting in splitting this genus into two different genera, based i.a. on the siphunculi state [47]. Six species have been transferred to a new genus *Archeoviparosiphum* Żyła, Homan, Franielczyk & Wegierek, 2015 with clearly porous siphunculi. Only one, characterized by longer siphunculi that is considered to be an apomorphic state, remained in *Oviparosiphum*. Moreover, the *Oviparosiphum* in a new sense is much younger than some other Oviparosiphidae genera because it is known from the Lower Cretaceous (Aptian) deposit—Bon—Tsagaan, Mongolia. Nonetheless, the Oviparosiphidae as a whole do not form a monophyletic group. The features, which are shared by all their taxa, could be the result of convergence.

### Systematic position of *Vitimaphis*

This genus is known from the Lower Cretaceous deposit—Baissa (Russia), and was originally described based on a single species. Here, we provide more data for the type species and also describe a new one. *Vitimaphis* is clearly separated from the other Oviparosiphidae by the combination of very short antennae and conical siphunculi. While one of the common features for the Bajsaphididae + *Vitimaphis* + Canadaphididae group is a very long segment III of antennae (12–2), the opposite state was recovered as an autapomorphy for *Vitimaphis* (12–0). In this case, this feature should be treated as a reversion for *Vitimaphis*. The only character that supports the monophyly of the Bajsaphididae + *Vitimaphis* + Canadaphididae is a well-developed anal plate (38). Since the position of the genus remains unclear, we are not making any taxonomic decision.

### Evolution of siphunculi

Traditionally, fossil aphids having both ovipositor and siphunculi have been assigned to the family Oviparosiphidae. For a long time, it was essentially the only group comprising aphids that possess both structures simultaneously. It has been considered as a transitional evolutionary stage between the older and modern aphid groups because the ovipositor is thought to be a plesiomorphic feature, while the siphunculi an apomorphic one. Most of extinct aphid families had only the ovipositor, which is uncommon for recent aphids. Now only representatives of the superfamilies Adelgidae and Phylloxeridae possess this structure [58]. All other recent aphids have only siphunculi that are much more structurally diverse than those among the fossil aphids. They vary greatly in length among recent taxa, from long cylinders to short cones, or even just pores. In some groups they bear setae or reticulations, which are used as a diagnostic feature. Although the function of siphunculi is already quite well known [59], it is still unclear why they vary in length among lineages. The main function of these structures is to secrete triglycerides, which act as a mechanical defence against natural enemies, and also alarm pheromones that induce behavioural responses in other individuals nearby [60]. Since siphunculi provide mechanical and chemical protection against enemies, and in recent aphids are usually associated with the colonial mode of living, the appearance of these structures is thought to be one of the key steps in the evolution of aphids. It has been hypothesized that the most primitive siphunculi are porous, and the progressive evolutionary trend was towards their elongation, e.g. [61]. However, both states are actually present within extinct aphids, even at the very early stage of their evolution—Middle/Late Jurassic. If Shaposhnikov’s hypothesis is correct, a common ancestor with the porous siphunculi probably must have been much older than currently thought, and obviously there were several lineages in which this character evolved independently. The polyphyletic Oviparosiphidae in our analysis is consistent with these assumptions.

### Aphid diversification

Three major adaptive radiation events have been proposed for aphid evolution: in the early Mesozoic, Late Cretaceous and around the Miocene [4, 5, 62]. All are thought to be a consequence of changes in the global vegetation, i.e. composition of the aphid host plants. Since the oldest aphids were associated with conifers, the occurrence of angiosperms could greatly influence their diversification. The most recent studies on insect diversification suggest that the rise of angiosperms did not generate an immediate increase in diversification within major insect groups, but the authors also pointed out that limitations of those analyses resulted from incomplete taxon sampling, and/or analytical methods. Another potential reason could be that angiosperm-associated insects first diversified on gymnosperm plant hosts, before initially shifting after the KTR with generalist angiosperm associations and later to specialist associations during the Late Cretaceous and Paleogene [63]. However, this hypothesis is inconsistent with observations of the extinct aphid fauna. Comparing aphid fossils from the Early Cretaceous to those from Late Cretaceous, we can observe faunal turnover, which may reflect the KTR. In this context a polyphyletic ‘Oviparosiphidae’ is not some ancestral lineage of the group that diversified later, but a trace of the group that was already diverse in the deep past, before the lineage turnover leading to modern aphids. Indeed, none of the Jurassic and Early Cretaceous groups survived, and only a few Late Cretaceous lineages gave rise to the recent fauna [8]. The next step should be to test this hypothesis in the analytical framework, but it requires a better understanding of the relationships between extinct and extant aphids.

## Conclusion and future directions

Our studies show that there is a great necessity for analytical reassessment of existing views on the taxonomic composition and sister-group relationships between extinct groups of aphids. We should go beyond traditional views because even such a relatively simple analysis as conducted here shows that they are not necessarily correct. Our analysis clearly showed that we should not treat Oviparosiphidae as a monophyletic group, and that there were more lineages with the concurrent presence of ovipositor and siphunculi than currently thought. For the time being, we are unable to make any taxonomic rearrangements of Oviparosiphidae since the fossil record is too sparse and it could cause unnecessary confusions. But more data on the Jurassic/Cretaceous aphids would help to distinguish their main lineages and clarify the status of taxa belonging to the Oviparosiphidae grade. This is especially important because it is still unclear which lineage should be considered as the stem lineage for all modern Aphidoidea. We decided to maintain the current status of the family and continue efforts to clarify the relationships between extinct aphid families. Also, having more resolved phylogeny of extinct aphids will allow assessing the state (plesiomorphic or apomorphic) of crucial morphological features, which in turn may help to understand the evolution of recent aphids and thus improve their classification.

## Supporting information

S1 File Data matrix in Nexus format. (NEX)
